# Emotional Complexity In Daily Life: On The Role Of Emotional Dynamics, Age, & Culture

**DOI:** 10.1093/geroni/igaa057.3386

**Published:** 2020-12-16

**Authors:** Yoonseok Choi, Jennifer Lay, Minjie Lu, Helene Fung, Christiane Hoppmann

**Affiliations:** 1 University of British Columbia, Vancouver, British Columbia, Canada; 2 University of Exeter, Exeter, England, United Kingdom; 3 Sun Yat-Sen University, Guangzhou, China; 4 The Chinese University of Hong Kong, Sha Tin, China

## Abstract

Emotional complexity is a construct that has attracted significant interest in the aging literature. It often refers to two aspects — the co-occurrence of positive and negative emotions and emotion differentiation (experiencing emotions with specificity). Emotional complexity is thought to increase with aging. However, recent research points to inconsistent results showing a positive relationship between age and emotional complexity, non-significant associations and even negative relationships. The present study seeks to address this inconsistency in findings by examining three possible sources: 1) different indicators of emotional complexity, 2) age differences in emotional dynamics (individual differences in means & variability of momentary positive & negative emotions), and 3) differences in cultural backgrounds. Community-dwelling adults from Vancouver (96 older adults, 51 young adults; 56% of Asian heritage, 30% of Caucasian heritage, and others 14%) and in Hong Kong (56 older adults, 59 young adults; 100% Asian heritage) completed approximately 30 ecological momentary assessments over a 10-day period assessing their current emotional experiences. When the mean and variability of emotional experiences were controlled for, most emotional complexity measures showed a negative relationship with age indicating that older adults displayed lower emotional complexity compared to young adults. This pattern was consistent across participants of Asian and Caucasian heritage. Additional analyses will explore the link between different emotional complexity measures and well-being indicators. Our findings point to the need to provide a more nuanced perspective on the correlates and consequences of emotional complexity in old age.

